# A Nanostructured Protein Filtration Device for Possible Use in the Treatment of Alzheimer’s Disease—Concept and Feasibility after In Vivo Tests

**DOI:** 10.3390/bioengineering10111303

**Published:** 2023-11-10

**Authors:** Thomas Gabriel Schreiner, Manuel Menéndez-González, Maricel Adam, Bogdan Ovidiu Popescu, Andrei Szilagyi, Gabriela Dumitrita Stanciu, Bogdan Ionel Tamba, Romeo Cristian Ciobanu

**Affiliations:** 1 Faculty of Medicine, University of Medicine and Pharmacy “Carol Davila”, 050474 Bucharest, Romania; 2 Faculty of Medicine, University of Medicine and Pharmacy “Gr. T. Popa”, 700115 Iasi, Romania; 3 Department of Electrical Measurements and Materials, Faculty of Electrical Engineering and Information Technology, Gheorghe Asachi Technical University of Iasi, 700050 Iasi, Romania; 4 Department of Medicine, University of Oviedo, 33006 Oviedo, Spain; 5 Department of Neurology, Hospital Universitario Central de Asturias, 33006 Oviedo, Spain; 6 Instituto de Investigación Sanitaria del Principado de Asturias, 33006 Oviedo, Spain; 7 Neurology Department, Colentina Clinical Hospital, 020125 Bucharest, Romania; 8 Laboratory of Cell Biology, Neurosciences and Experimental Myology, ‘Victor Babes’ National Institute of Pathology, 050096 Bucharest, Romania; 9 Advanced Research and Development Center for Experimental Medicine (CEMEX), “Grigore T. Popa” University of Medicine and Pharmacy, Universitatii Str., No. 16, 700155 Iasi, Romania

**Keywords:** Alzheimer’s disease, neurodegenerative disease, nanoporous ceramic filter, protein filtration, cerebrospinal fluid, scanning electron microscopy, biocompatibility

## Abstract

Background: Alzheimer’s disease (AD), along with other neurodegenerative disorders, remains a challenge for clinicians, mainly because of the incomplete knowledge surrounding its etiology and inefficient therapeutic options. Considering the central role of amyloid beta (Aβ) in the onset and evolution of AD, Aβ-targeted therapies are among the most promising research directions. In the context of decreased Aβ elimination from the central nervous system in the AD patient, the authors propose a novel therapeutic approach based on the “Cerebrospinal Fluid Sink Therapeutic Strategy” presented in previous works. This article aims to demonstrate the laborious process of the development and testing of an effective nanoporous ceramic filter, which is the main component of an experimental device capable of filtrating Aβ from the cerebrospinal fluid in an AD mouse model. Methods: First, the authors present the main steps needed to create a functional filtrating nanoporous ceramic filter, which represents the central part of the experimental filtration device. This process included synthesis, functionalization, and quality control of the functionalization, which were performed via various spectroscopy methods and thermal analysis, selectivity measurements, and a biocompatibility assessment. Subsequently, the prototype was implanted in APP/PS1 mice for four weeks, then removed, and the nanoporous ceramic filter was tested for its filtration capacity and potential structural damages. Results: In applying the multi-step protocol, the authors developed a functional Aβ-selective filtration nanoporous ceramic filter that was used within the prototype. All animal models survived the implantation procedure and had no significant adverse effects during the 4-week trial period. Post-treatment analysis of the nanoporous ceramic filter showed significant protein loading, but no complete clogging of the pores. Conclusions: We demonstrated that a nanoporous ceramic filter-based system that filtrates Aβ from the cerebrospinal fluid is a feasible and safe treatment modality in the AD mouse model. The presented prototype has a functional lifespan of around four weeks, highlighting the need to develop advanced nanoporous ceramic filters with anti-biofouling properties to ensure the long-term action of this therapy.

## 1. Introduction

The incidence and prevalence of central nervous system (CNS) disorders, especially neurodegenerative diseases (NDDs), are continuously increasing, with predictions suggesting a doubling in their related figures in the next decades [1]. Particularly, Alzheimer’s disease (AD) represents a significant socioeconomic burden, with devastating consequences for the affected patients and their relatives, and high costs for healthcare systems [2].

There are a couple of main reasons for why AD is still a challenge for clinicians despite the huge number of already-conducted clinical studies [3]. Firstly, despite many intensely debated theories, such as the amyloid hypothesis [4], the neuroinflammatory theory [5], the impact of oxidative stress [6], or the role of ion metals in neurodegeneration [7], the etiology of this disease remains incompletely known up to the present. Yet, excessive brain accumulation of the amyloid beta protein (Aβ) via the amyloidogenic pathway is considered the main pathological hallmark of AD [4]. Moreover, in late-onset AD, which represents almost 95% of the total AD cases worldwide, Aβ pathological aggregation in the brain is considered to be the result of an impaired CNS elimination, rather than the consequence of excessive production [8]. Thus, cerebral and peripheral Aβ found in different forms such as monomers, oligomers, and senile plaques is a frequent target for drug-based therapeutic approaches, the proof of this being the anti-Aβ monoclonal antibodies Aducanumab [9] and Lecanemab [10], which were recently approved for clinical use.

Secondly, because of anatomical aspects such as the highly selective blood–brain barrier (BBB), drug delivery to the brain and other minimal invasive therapeutic methods raise implementation difficulties and are correlated with high rates of adverse effects [11]. In normal conditions, the BBB plays a protective role for the CNS, limiting the entrance of toxic molecules from the systemic circulation [12]. However, it only allows 5–10% of drugs to cross towards the cerebral matter, with drug size and solubility the leading causes of this low BBB penetrance [13]. Because of the lack of effective technologies to deliver drugs to the brain via the BBB, novel strategies should be explored to develop efficacious anti-dementia therapies.

In this context, intraparenchymal drug delivery might be considered an option, together with the intrathecal route, based on the close connection between the cerebral parenchyma and the cerebrospinal fluid (CSF) [14]. However, as the administration of drugs directly into the brain tissue or CSF is associated with significant side effects, indirect approaches, such as the pseudo-delivery of medication at the CSF level or the filtration of the CSF through specialized devices, could be valuable options in the near future.

In this paper, we propose an alternative way to remove Aβ from the interstitial fluid (ISF) by increasing its clearance directly from the CSF based on the “Cerebrospinal Fluid Sink Therapeutic Strategy”, a theory first developed by the authors in prior publications [15,16]. According to this hypothesis, there is an equilibrium between the soluble Aβ in the CSF and the Aβ in the ISF; therefore, the removal of Aβ from the CSF promotes the efflux of the Aβ from the ISF. Reducing the load of soluble Aβ from the brain leads to a reduced accumulation of Aβ in the form of insoluble Aβ plaques with the restoration of cerebral Aβ dynamics, as well as a subsequent reduction in Aβ production and improved clearance. From a clinical point of view, this therapeutic concept would reduce the progression rate of AD and delay its clinical onset.

In order to achieve the selective clearance of Aβ from the CSF, intrathecally implantable devices are needed. Drug delivery systems (DDSs) based on nanoporous ceramic filters are of great interest because the physicochemical characteristics of the pores can be tailored to control the rate of drug delivery or protein separation; however, they have the disadvantage that they are prone to suffering biofouling, thus limiting their performance. The ultimate goal of this original article is to confirm the feasibility of this new therapeutic approach by using an experimental nanoporous ceramic filter as a CSF filtration device for the potential treatment of AD and other NDDs where there is an accumulation of proteins in the brain. Thus, in the first part, the authors summarize the main steps needed to create a novel CSF filtration device, focusing on the synthesis, functionalization, quality control, and biocompatibility testing of the alumina nanoporous ceramic filter. The desired Aβ-selective filtration is achieved via a two-step process: firstly, the alumina nanoporous ceramic filter ensures the size-based filtration of small proteins (including Aβ monomers and oligomers) located in the CSF, and secondly, based on the highly specific antigen–antibody reaction, only the Aβ compounds are sequestered and definitively eliminated from the CSF with the help of anti-Aβ antibodies or Neprilysin–Albumin. In the second part, the prototype is inserted in an AD mouse model for four weeks, with subsequent explantation to assess the state of the nanoporous ceramic filter after in vivo use.

## 2. Materials and Methods

### 2.1. Development of the Nanoporous Ceramic Filter

The development of an alumina nanoporous ceramic filter suitable for the selective Aβ filtration from the CSF is a complex, multi-step process that includes the following phases: synthesis, functionalization, and quality control of the functionalization, which can be executed using various methods, selectivity measurements, and an assessment of its biocompatibility. While there are commercially available anodized alumina oxide (AAO) ceramic filters, their large pore sizes and different pore densities and distributions make them imperfect candidates as performant nano sieves. In this regard, we chose to develop an efficient Aβ-filtrating nanoporous ceramic filter whose function is based mainly on protein size exclusion.

According to the literature and previous experience of the authors, the optimal method for the synthesis of a highly qualitative filtrating nanoporous ceramic filter is a two-step anodizing process using oxalic acid-based electrolytes. The detailed steps for transforming aluminum disks into alumina nanoporous ceramic filters are described in another previous work by the authors [17].

Subsequently, the functionalization of the alumina structure is assured via the atomic layer deposition (ALD) technique, resulting in a two-layer coating of a 4 nm thickness.

According to the functionalization of the nanoporous ceramic filter, the authors could manually fine-tune the desired ceramic filter parameters within strict limits. This allows the possibility of adapting the non-functionalized nanoporous ceramic filter for selective molecular permeability, concerning both the molecular target from the fluid (i.e., Aβ monomers and oligomers) and the drug that is to be used (anti-Aβ antibodies or Neprilysin–Albumin), in order to bind the molecular target.

The next crucial step is the quality control of the functionalized ceramic filter. This can be carried out in several ways, using techniques such as X-ray diffraction, Raman spectroscopy, infrared spectroscopy, scanning electron microscopy (SEM) with energy dispersive X-ray spectroscopy (EDS), and a simultaneous thermal analysis (consisting of thermogravimetry and differential scanning calorimetry).

In our case, preliminary morphological analyses of the surface (both the top/apical and bottom/basal surfaces) of the nanoporous ceramic filter samples were performed via SEM analysis. Cross-sectional images obtained after the mechanical fracturing of the nanoporous ceramic filter were also obtained. To ensure proper contrast and the required electrical conductivity, the fractured samples were covered with a thin layer of gold (Au) using spray deposition before testing. Chemical characterization of the uncoated and ALD-coated alumina ceramic filters and compositional profile scans, allowing the determination of the ALD-deposited layers’ elemental distribution along the nanoporous ceramic filter’s pores, were performed via local X-ray diffraction analysis.

The diffractogram of the sample was recorded with a D8 Advanced X-ray diffractometer from Bruker using CuKα radiation (λ = 1.54056 Å). Raman and IR spectra of the sample were recorded with a Fourier transform (FT) Raman spectrophotometer, model MultiRam, and a Fourier transform infrared (FTIR) spectrophotometer, model Vertex 80, both equipment having been purchased from Bruker.

The primary concern when developing a nanoporous ceramic filter to filtrate Aβ from the CSF is its selectivity. Thus, the customized nanoporous ceramic filter should be permeable for Aβ monomers (and small oligomers), but impermeable for other molecules found in the CSF, such as albumin. The preliminary characteristics of our desired nanoporous ceramic filter, related to its function of Aβ filtration, are the following: maximum diameter of 10 mm, thickness of 50–60 μm, pore size of 10 nm, and interpore distance from 30 to 100 nm. Potential measurements of the preliminary ceramic filter samples were conducted using a test cell consisting of two glass half-cells, a magnetic stirrer at the bottom of each half-cell to minimize the effect of polarization on the ceramic filter surfaces, and two Ag/AgCl reversible electrodes placed in each half-cell and connected to a digital voltmeter. This measuring technique is also presented in detail in our previous study [17]. According to the results, Aβ is in complete equilibrium on both sides after 72 h, while albumin does not cross the nanoporous ceramic filter. Despite us not directly measuring the Aβ diffusivity of the functionalized nanoceramic filter, the same concentration of Aβ in both half-cells confirmed its complete permeability (100%).

Finally, biocompatibility tests should be conducted after ensuring that the proposed nanoporous ceramic filter falls within the desired parameters. In this sense, we investigated in vitro the biocompatibility of nanoceramic filter samples on two distinct cell cultures (MCF-7 and MDA-MB-231 cells) using an MTT assay. This method is based on quantifying living (and dead) cells after incubation of the sample (in different concentrations) after 24, 48, and 72 h. Only after successfully passing all these steps was the nanoporous ceramic filter used as the experimental device’s main filtrating component.

### 2.2. Development of the CSF Filtration Device

**Figure 1:**
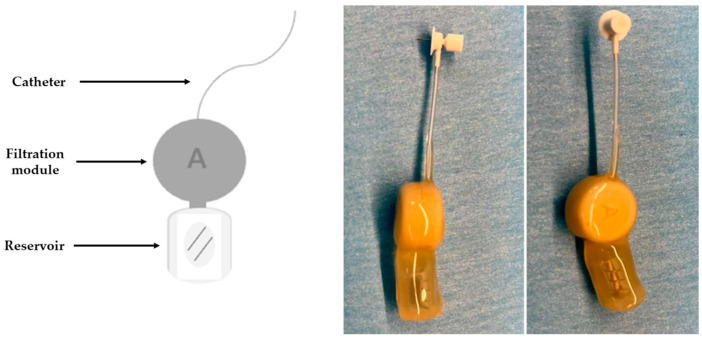
Schematic representation (**left**), lateral view (**middle**), and superior view (**right**) of the CSF filtration device.

**Figure 2:**
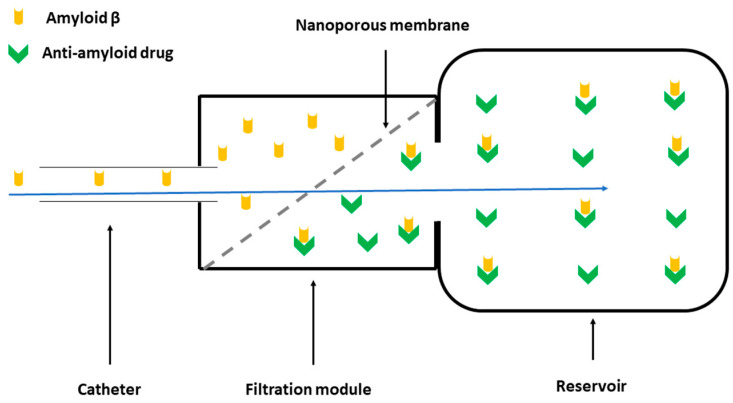
Schematic representation of the filtration module: the Aβ-rich CSF travels through the nanoporous ceramic filter (blue arrow), where the size-based filtration takes place; subsequently, via a specific antigen–antibody reaction, Aβ is permanently sequestered in the reservoir via the anti-Aβ drugs.

The CSF filtration device’s assembly becomes easier after the biocompatible nanoporous ceramic filter has been completed. As seen in Figure 1, the prototype consists of three main parts: the catheter, the filtration module, and the reservoir. The catheter is the longest part of the assembly, connecting the lateral ventricle to the filtration module. The most relevant part of the filtration module is represented by the nanoporous ceramic filter, which has an essential role in separating Aβ from the CSF. Figure 2 is the filtration module’s schematic representation, highlighting both this part’s main components and its operating principle. Finally, the reservoir is a single-unit closed chamber where Aβ is bonded to albumin and subsequently sequestered from the CSF. Because of the nanoceramic filter’s high selectivity, unbound Aβ can pass bidirectionally through the nanoceramic filter between the filtrating module and the reservoir; however, albumin-bound Aβ remains in the reservoir.

The capsule of the filtrating module and the covers protecting the whole device are made of polyetheretherketone (PEEK), a thermoplastic polymer suitable for our experiments because of its resistance and mechanical strength, very low moisture uptake, and good dimensional stability. The assembly consisting of the filtrating module has the following parameters: height of approx. 3.7 mm, width of 1 mm, and diameter of 12 mm. Regarding the reservoir, this part is made of silicone, has the biggest dimensional parameters (2 cm length, 1 cm width, and 0.7 cm height), a septum with a 180° access opening, and a total volume of 100 µL. The catheter, a simple tube, connects the filtrating module and the reservoir with the ventricular space through an additional elbow-shaped part at its tip. The polycarbonate elbow is a 30G stainless steel tube (ID: 0.16 mm; OD: 0.31 mm; depth: adjustable from 1 to 3 mm with a polycarbonate spacer 0.5 mm high from the surface), attached to the catheter via a 0.71 mm (21G) stainless steel tubing. The catheter tube material is made of medical-approved polyvinyl chloride and has the following parameters: 15 cm length, 0.69 ± 0.08 mm ID, 1.14 ± 0.08 mm OD, 56.23 µL total volume (56 µL from the 15 cm catheter and 0.23 µL from the 3 mm stainless steel tubing).

In order to have all components fixed in a one-piece unit, silicon adhesives were used. We preferred the use of several adhesives according to their characteristics and the characteristics of the parts that needed to be glued. For example, we used Loctite^®^ SF 7701™ for difficult-to-bond substrates, such as PEEK, and also because the prototype was designed as a single-use medical device. In order to ensure a stronger bond, we mixed the Loctite Primer with Loctite^®^ 4061, a clear, colorless, low-viscosity instant adhesive that is particularly suitable for plastics, rubber, and biological components [18]. In the final steps of the production, Loctite^®^ SI 5248 was used. This translucent, yellow, high-strength, high-flexibility silicone-based adhesive is suitable for many substances and was used to create a soft and biocompatible device coupling and protect its connections.

### 2.3. Device Implantation on Mice

In order to assess the validity of the Aβ filtration concept, the next step of our research was to implant the experimental device in a mouse model simulating an AD patient. One group of 5 APP/PS1 mice received surgically implanted devices for a period of 4 weeks. The animals weighed at least 25 g and were older than 35 weeks; these parameters are essential because of the device dimensions and because, in this specific AD model, the formation of Aβ plaques and the onset of cognitive deficit starts at the age of 7 months [19]. The implantation procedure can be separated into three different steps: presurgical preparation, device placement, and post-surgical supervision, according to previous similar experiments conducted in the literature [20,21].

The presurgical preparation consists of filling the reservoir of the device under sterile conditions, with the therapeutic agent/drug dissolved in the vehicle solution for all five animals. Additionally, before initiating the surgical procedure, it is mandatory to confirm that the catheter is not blocked.

Before the placement of the device, the animal must be anesthetized through inhaled anesthesia with isofluorane (3%) and oxygen. Subsequently, the mouse is placed in the stereotaxic frame in order to make the presurgical measurements. After exposing the surface of the skull and executing a subcutaneous pocket starting from the base of the neck towards the hindlimbs (appropriate for the size of the device), the next step implies finding the correct puncture coordinates for the right lateral ventricle. In this sense, after locating the bregma, the optimal cannula placement point is 1.25 mm on the left–right axis and 0.7 mm on the anterior–superior axis, similar to previous data in the literature [21]. After the trepanation process, the cannula is inserted, and simultaneously, the device is introduced into the subcutaneous pocket. Once the CSF filtration device is mounted, a simple suture is required as the final step of the surgical procedure.

**Figure 3:**
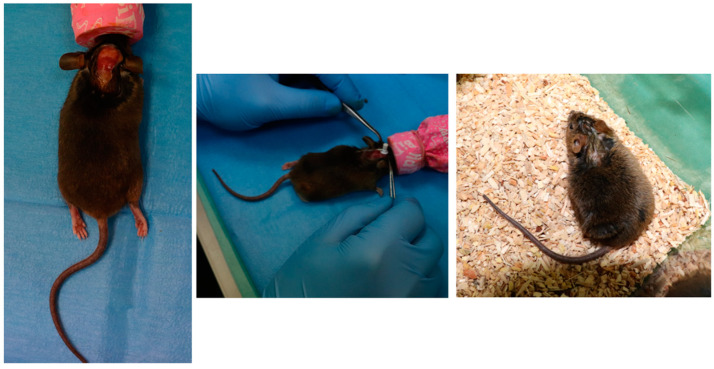
Implantation of the device in the AD mouse model (**left**—exposure of the surface of the anesthetized mouse’s skull, **middle**—cannula insertion, **right**—post-surgical monitoring).

Finally, at the end of the surgery, after the mouse awakens, meloxicam (2 mg/Kg) is administered subcutaneously, and 1 mL saline solution (NaCl-0.9%) is injected intraperitoneally. The first 24 h after surgery are critical, so the mice were kept in intensive care conditions, with controlled temperatures (28–30 °C), and easy access to food and water. Post-surgical medication was administered for three days after surgery: buprenorphine (0.05 mg/Kg) subcutaneously every 12 h, and meloxicam (2 mg/Kg) subcutaneously every 24 h. Figure 3 depicts the most important steps of the surgical implantation of the devices.

All of the procedures conducted on animals were approved by the ethics committee of the University of Medicine and Pharmacy “Gr. T. Popa”, Iasi, Romania, in line with the general principles of the Helsinki Declaration of 1975, revised in 2008, and the current European legislation.

### 2.4. Nanoporous Ceramic Filter Assessment after a 4-Week Trial Period

After a 4-week trial period, the devices were explanted from the mice, cleaned, and broken down into their main components. The nanoporous ceramic filters were taken and retested for their accumulation of organic materials using two complementary techniques: SEM analysis, and a simultaneous thermal analysis (thermogravimetric/differential scanning calorimetry), respectively.

SEM images are produced as a result of the interaction between the electrons of the emergent beam and the atoms located at various depths in the tested sample [22]. Via this process, several types of signals are produced, such as secondary electrons, reflected electrons, transmitted electrons, and cathodoluminescence. Most SEM equipment (including the machine used in our experiments) only has detectors for secondary electrons. Because of their very low energy levels (50 eV), these secondary electrons have a limited free path in solid substances, thus they escape a few nanometers from the sample’s surface and are localized at the impact point of the primary beam [23]. This ensures the production of high-resolution (below 1 nm) images.

Thermogravimetry (TG) is a method of thermal analysis based on the measurement of variations in a sample’s mass according to temperature changes over time [24]. Via TG, a sample can be characterized according to its physical phenomena, such as transition, adsorption, or absorption, or chemical phenomena, such as thermal decomposition, chemical adsorption, or solid–gas reactions. Differential scanning calorimetry (DSC), on the other hand, delivers information on the heat flow of a specific sample as a function of temperature over time [25].

The apparatus used in our experiments, a simultaneous thermal analyzer NETZSCH STA 449 F3 Jupiter^®^, located in the endowment of INCDIE ICPE-CA, within the Department of Metallic, Composite, and Polymeric Materials, combines the principles of these two measurement methods. This equipment uses a wide temperature range (−170 °C to 700 °C) and is intended for inorganic products for the determination of their purity, as well as their thermal stability, mass loss, and the onset of the decomposition process. The recorded curves were processed using the Calisto Processing software version 2.13 provided by the equipment manufacturer to determine the characteristic parameters of the analyzed material: melting temperature (Tt), melting enthalpy (Ht), and oxidation start temperature (OOT).

## 3. Results

### 3.1. Development and Characterization of the Functionalized Nanoporous Ceramic Filter

**Figure 4:**
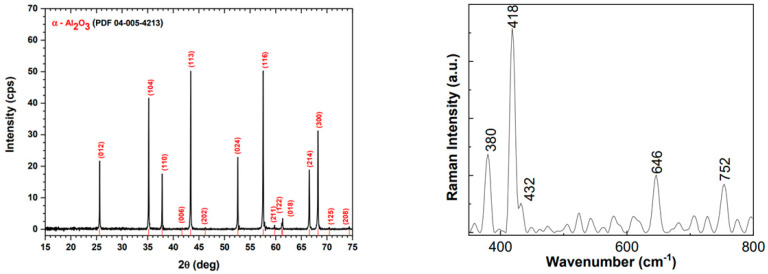
X-ray diffraction pattern (**left**) and Raman spectroscopy (**right**) of the developed nanoporous ceramic filter.

In order to assess the correct production and functionalization of the alumina ceramic filter, several steps are required, as detailed in the previous section. Morphological and chemical features of nanoceramic filter samples were preliminarily analyzed using X-ray diffraction, infrared, and Raman spectroscopy. Similar to the results from our previous work [17], the X-ray diffraction pattern of the sample showed peaks at 2θ diffraction angles corresponding to the crystalline planes of the crystallized α-Al_2_O_3_ in a rhombohedral crystal system. In line with other reports in the literature [26], reviewing these peak positions, we can conclude that the alumina sample was very well crystallized, showing narrow and sharp X-ray diffraction peaks. Similarly, Raman spectroscopy showed several lines assigned to different vibrational modes that are characteristic of α-Al_2_O_3_ (Figure 4). The infrared bands peaked at specific wavelengths which were also highly suggestive of the vibrational modes of Al–O stretching in the octahedral structure, O–H deformation in Al–O–H, aged α-Al_2_O_3_, Al=O, and α-Al_2_O_3_ [27]. All of these complementary measurements demonstrate the successful functionalization of the alumina ceramic filter via the ALD technique.

**Figure 5:**
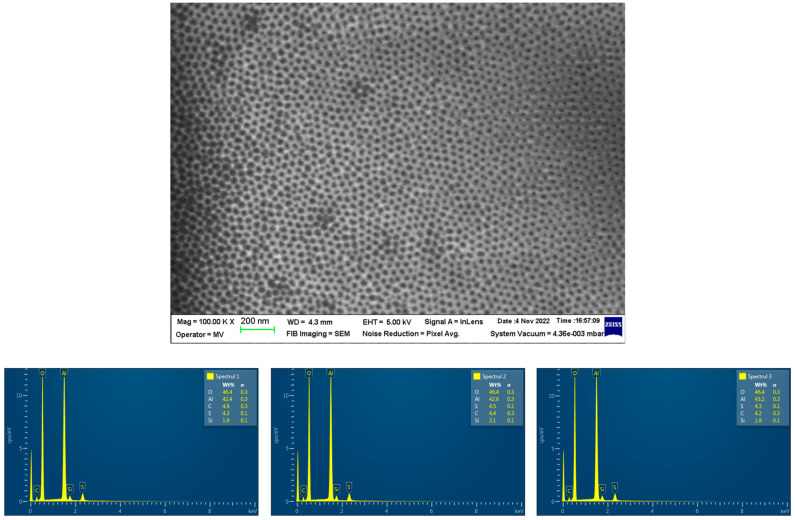
SEM (**upper row**) and EDS (**lower row**) analysis of the nanoporous ceramic filter before implantation (magnification of 100,000×).

The morphological and chemical changes in the sample pores associated with the ALD functionalization treatment were analyzed using SEM (and local energy dispersive X-ray spectroscopy—EDS) (Figure 5). The image at 100,000× magnification indicates the beginning of amorphization (the contours become blurred as the beam sweeps over the surface of the sample), explained by the fact that little residual carbon is present on the surface. The surface of the analyzed material is non-conductive, which led to a loss of contrast and resolution of the highlighted structure. For the acquisition of these images, the charge compensation method via nitrogen gas flow was used.

It is observed that the analysis of several areas of the EDS spectrum (in this presentation, at least three; see Figure 5) showed high reproducibility of the ALD process: respectively, very low variability of O (below 1.7%, with an average of 46.12%) and Al (below 2.5%, with an average of 42.86%). In this process, carbon (C) and sulfur (S) appear in very small and controllable percentages (on average below 4.5%), respectively, with traces of phosphorus (P) and silicon (Si).

An alpha-aluminous material was most often found in the SEM analysis, similar to the Raman spectroscopy, the majority being the Al_2_O_3_ phase composed of the elements identified using EDS: Al and O, with traces of SiO_2_.

**Figure 6:**
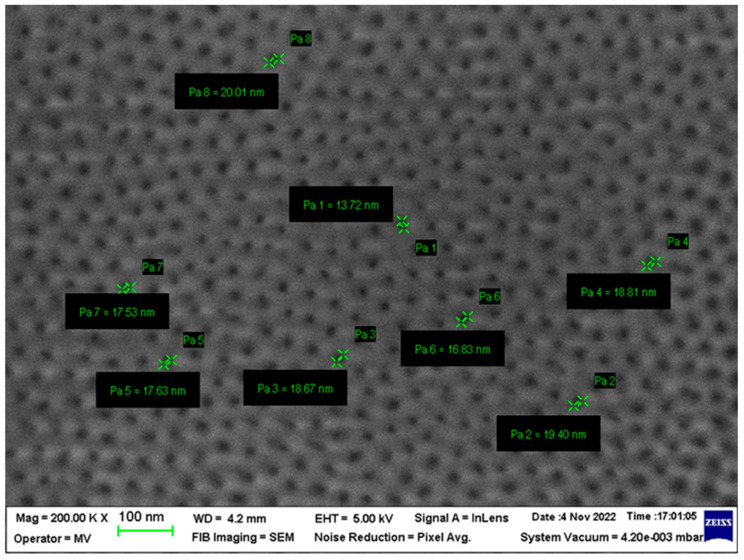
SEM analysis of the nanoporous ceramic filter before implantation—magnification of 200,000×. Pores were able to be visualized and automatically measured.

Due to their nanometric size, the pores appear visible only at magnifications above 50,000×. However, greater SEM/EDS magnifications, up to 200,000×, are needed for optimal pore analysis, as depicted in Figure 6. The highlighted pores are not communicating and are perpendicular to the actual filter section and have, at a first evaluation, an x–y dimension of approx. 13–19 nm after ALD amorphization. An initial analysis of the distribution of the pores shows us their uniform distribution and the quasi-equal sizes of the diameters of the pores (Figure 6).

Through the preliminary design of the ideal technology, we proposed the following parameters: pore size of up to 18.00 nm, maximum pore size dispersion of approx. 2 nm, and interpore space up to 110 nm. According to the results of the abovementioned measuring techniques (mainly SEM and EDS), remarkable parameters were obtained: pore size of approx. 17.00 nm, maximum pore size dispersion of approx. 2 nm, and an interpore space from 35 to 50 nm. Table 1 summarizes the most relevant parameters of the developed nanoporous ceramic filter, including its thickness and molecular weight cut-off (MWCO).

It can be seen that the technological process (mainly the ALD process) was very well conducted, as the final product pores had an average size of approx. 17 nm, ideal for filtering Aβ, which requires pores below 25 nm (ideally below 20 nm). This dimension was considered according to the work of Kim et al. [28], where a required height of 2–3 nm and width of 5–25 nm for Aβ oligomers is stated. Spherical Aβ aggregates have diameters ranging from 15 to 35 nm and may have a width of 200–400 monomers. In comparison, fibrillary forms have typical diameters of over 10 nm and can grow to several micrometers in length. Alternatively, in our work, for increased efficacy, we opted for Aβ monomer and small oligomer size-based filtration.

In addition, it can be observed that, through controlling the anodizing process, the obtained pores were much more uniform and spatially closer than initially considered in the theoretical design, i.e., an interpore space of less than 50 nm. The direct impact of this was an increase in the efficiency of filtration, and also, because it was found that a smaller filter surface could be sufficient, additional miniaturization of the device could be considered.

**Figure 7:**
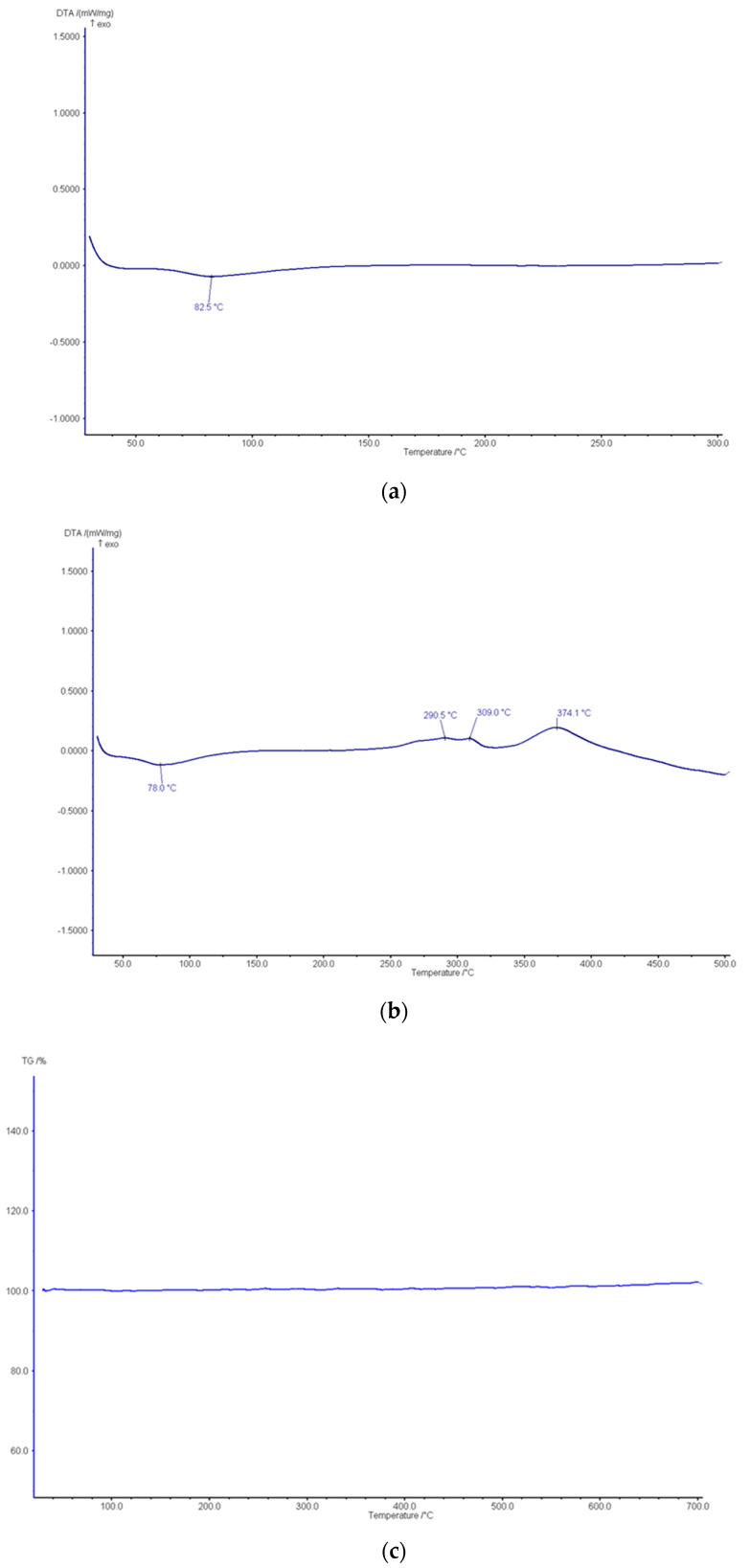
(**a**): DSC analysis of the nanoporous ceramic filter in nitrogen up to 300 °C. (**b**): The DSC analysis of the nanoporous ceramic filter in air up to 500 °C. (**c**): The TG analysis of the nanoporous ceramic filter in nitrogen up to 700 °C.

Further, we performed TG/DSC analysis of the final product at different temperatures and in several media. Initially, DSC analysis was performed in nitrogen up to 300 °C in order to observe if there was any organic/polymeric contamination, an essential aspect of filters designed for the separation of organic molecules. Only a weak endothermic process was observed at around 82.5 °C, probably due to the elimination of residual moisture; otherwise, this characteristic is specific to uncontaminated inorganic materials (Figure 7a).

In the results of the DSC analysis in air up to 500 °C, there is evidence of some weak exothermic processes from 290 °C to 375 °C, explained by the oxidation of traces of C and/or P (Figure 7b). A weak endothermic peak is observable at around 78 °C, probably correlated to the endothermic peak at 82.5 °C in nitrogen, as mentioned before.

Despite several peaks in the DSC analysis results (both in air and in nitrogen), for the TG analysis in nitrogen up to 700 °C, nothing statistically significant was observed (straight line). This does not exclude a very weak (unnoticed) weight loss due to the evaporation of small amounts of water or loosely bonded organic material. However, these results reconfirm the specific characteristics of inorganic materials uncontaminated with organic parts within the nanoceramic filter’s structure (Figure 7c).

**Figure 8:**
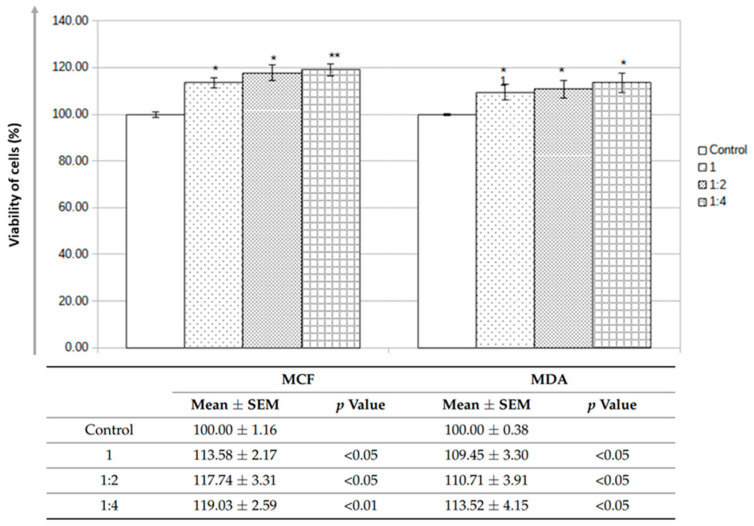
Results of the biocompatibility tests conducted on MCF-7 and MDA-MB-231 cells for different concentrations of nanoceramic filter sample extracts after 48 h; * denotes *p* < 0.05; ** denotes *p* < 0.01.

Lastly, the biocompatibility of nanoceramic filter samples was tested according to the MTT assay protocol. Two distinct cell cultures (MCF-7 and MDA-MB-231 cells) were used, their viability being quantified after incubation with the sample (in different concentrations) for 24, 48, and 72 h. The alumina nanoceramic filter did not influence the viability of the MCF-7 cells; on the contrary, an increase in the cell number/viability was observed with respect to the dilution of the sample extract. In the case of the MDA-MB-231 cells, the trend was similar, with the nanoceramic filter sample extracts being associated with an increased number of viable cells (Figure 8). Statistical calculations (mainly the student’s *t*-test) demonstrated statistical relevance in both cases.

### 3.2. Analysis of the Nanoporous Ceramic Filter after the 4-Week In Vivo Treatment

The preliminary analysis of the biocompatible nanoporous ceramic filter demonstrated their high quality and purity, permitting their use as the central component within the CSF filtration device. After developing a size-adapted prototype for our AD mouse model, the implantation of the device in five mice followed the steps presented in Section 2.3. The surgical procedure had a 100% survival rate, with no significant periprocedural or postprocedural adverse effects. During the 4-week treatment period, daily animal monitoring was conducted; one mouse (20%) needed surgical correction of the dehiscent skin flaps near the initial area of the cannula implantation. No infections, general status alterations, or other negative impacts of the CSF filtration device were noted during treatment. After the 4-week trial period, the animals were sacrificed, the devices were explanted and disassembled, and the nanoporous ceramic filters were taken for re-analysis.

Firstly, a combined SEM and EDS analysis was conducted, based on the same protocol applied before the device implantation. The five samples were examined on both sides, revealing the surfaces with a greater amount of organic material deposits and the cleaner surfaces with fewer deposits. To increase the clarity and relevance of the results, the following aspects should be considered: the surface of the nanoporous ceramic filter that first (directly) made contact with the Aβ-rich CSF is considered the top/apical surface, and the other surface of the nanoporous ceramic filter is considered the bottom/basal surface. The results presented below focus on the most relevant individual aspects and represent the final results obtained after statistical analysis of all five samples, which were similar in protein accumulation and clogging.

**Figure 9:**
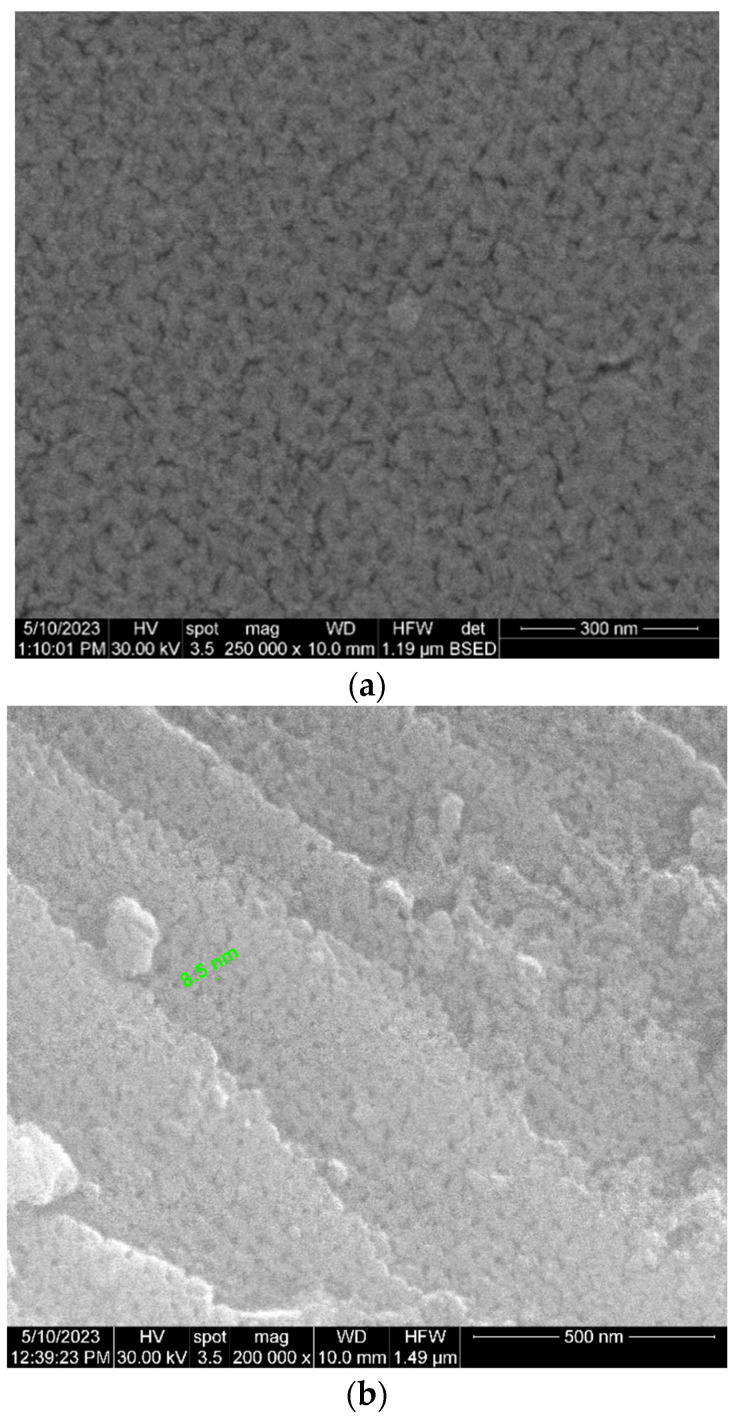

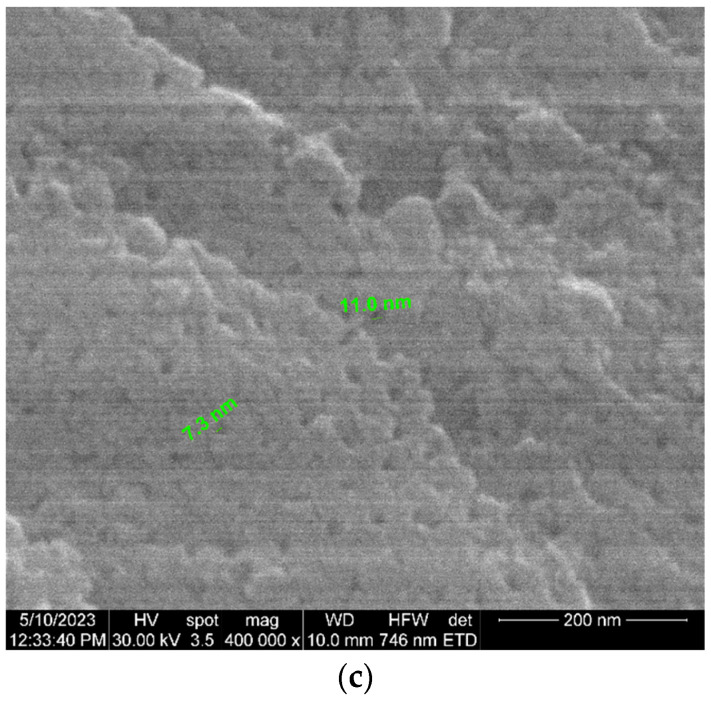
SEM analysis of the nanoporous ceramic filter after the 4-week treatment trial: (**a**) basal surface, magnification of 250,000×; (**b**) apical surface, magnification of 200,000×; (**c**) apical surface, magnification of 400,000×.

Comparing the two surfaces of the nanoporous ceramic filters, the SEM examination revealed the existence of a cleaner surface with fewer deposits on the basal surface, with a clear pore identification (Figure 9a). The apical surface showed a more significant amount of organic material deposits, disposed irregularly on the surface of the ceramic filter (Figure 9b). Comparing both sides of the ceramic filters, it is evident that the pores may be identified as still functional, but that the diameters of the pores diminished in both cases, with the most radical reduction noticed in the filtration area. At very high magnifications (400,000×), the porous structure of the studied ceramic filter can still be clearly distinguished, with the pores partially clogged with organic debris. In measuring the diameter of the pores on the apical surface, a reduction of about 50% of the initial diameter can be counted (an average of 8.5 nm compared to the original 17 nm diameter) (Figure 9b,c). Consequently, after the 4-week treatment trial period, the estimation revealed that the ceramic filter kept its filtration features and the pores were open (not obturated with organic matter), but the ceramic filter efficiency was reduced to a maximum of 25%.

**Figure 10:**
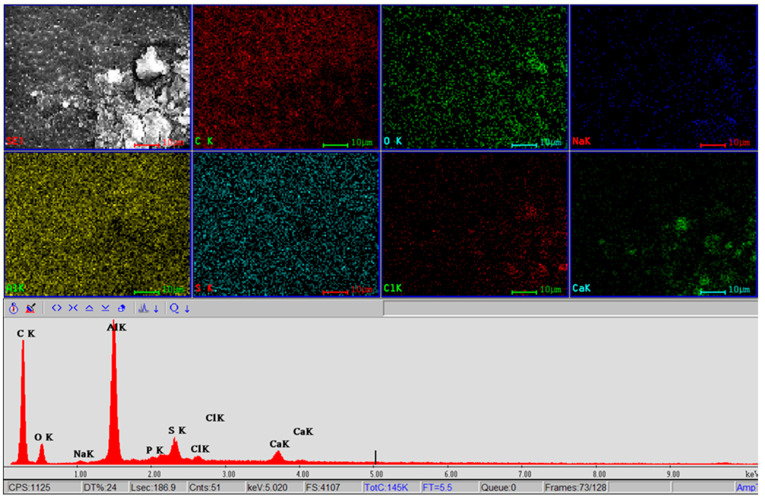
SEM and EDS analysis of the apical area of the nanoporous ceramic filter after the 4-week treatment trial—general mapping.

The general mapping of the filtration area obtained via joint SEM and EDS analysis after the 4-week treatment trial can be seen below in Figure 10. The presence of Al and O elements originating from the alumina ceramic filter was found. Still, some new peaks recorded via EDS with different intensities were directly proportional with the load of organic and some inorganic substances located on the surfaces of the samples, e.g., the existence of spectral lines for Al and O, but also C, Na, Cl, Ca, Au, Si, S, and P, can be noted.

Na and Cl elements originate from a saline solution specific to the filtered liquid, and Ca and P are related to protein residues. Sulfur (S) can come from the decomposition of some proteins into water-soluble fragments. The Au element comes from the preparation of the sample for electron microscopy analysis. As a conclusive result of the general mapping of the surface corresponding to the SEM image, the organic residues are more enriched with C, Ca, and O, and, to a lesser extent, with Na and Cl.

**Figure 11:**
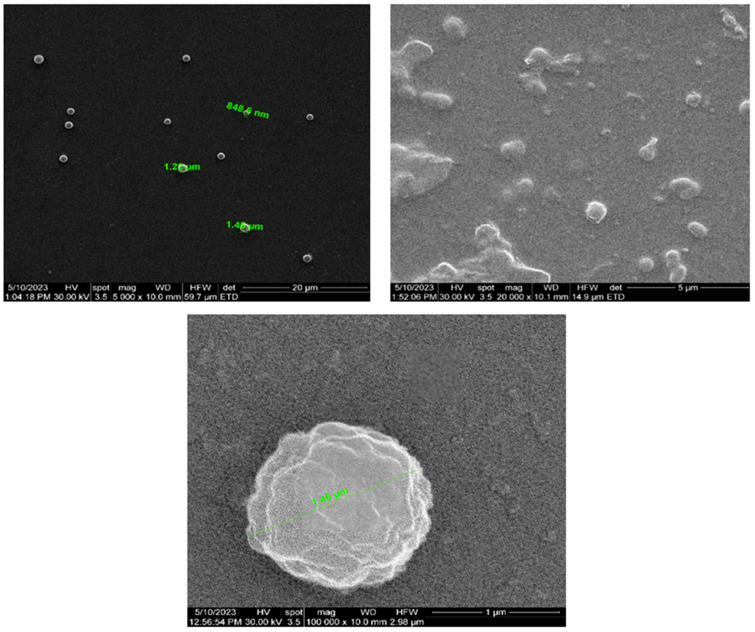
SEM analysis of the apical surface of the nanoporous ceramic filter after the 4-week treatment trial—comparative results of clogged debris versus ceramic filter with uniform distribution of filtered matter (magnifications of 5000×, 20,000×, and 100,000×).

On the other hand, SEM images also detected the existence of some round formations (clogged debris), specifically for spherical and/or fibrillar Aβ aggregates with large irregular surfaces, of approx. 1–1.5 µm (Figure 11). The detection of such large organic debris was expected in the context of Alzheimer’s disease and fully justifies the use of the proposed CSF filtration prototype, according to the Cerebrospinal Fluid Sink Therapeutic Strategy [15,16].

**Figure 12:**
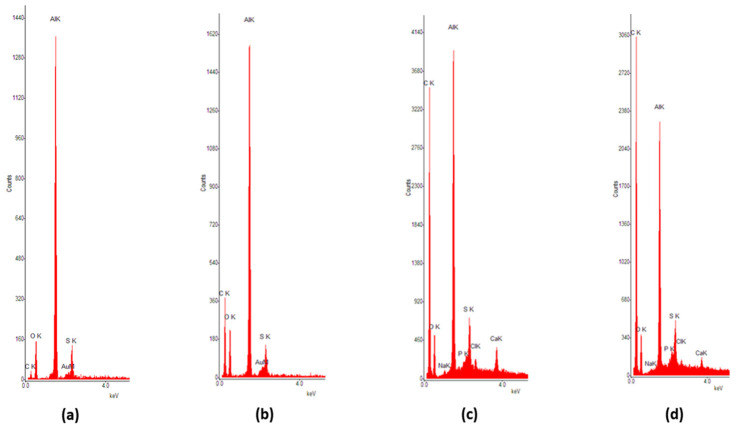
EDS analysis of the nanoporous ceramic filter: (**a**) before the 4-week treatment trial; (**b**) basal surface after the 4-week treatment trial; (**c**) apical surface containing uniform deposition of organic matter; (**d**) apical surface with large organic aggregates and complete clogging of the pores.

Regarding the SEM analysis highlighted in Figure 11, larger depositions and partial ceramic filter clogging with protein aggregates can be noticed. Although complete clogging of the pores is only observable in those small areas, the ceramic filter remains active in the unclogged parts, even if its efficiency is markedly reduced. This tendency is also supported by the results of the EDS analysis. Figure 12 and Table 2 highlight the results of the EDS analysis of the nanoporous ceramic filter before use (Figure 12a) and after the 4-week treatment trial, when several areas of the nanoporous ceramic filter were examined: the basal surface (Figure 12b); the apical surface, containing a uniform protein deposition (Figure 12c); and the large aggregate of organic matter on the apical area, which resulted in the complete clogging of the pores (Figure 12d).

The elements Na and Cl from the filtered saline solution present a low increase, being attached to organic matter remnant at the filtering surface. The highest growth in the intensity of the peaks is observed for C, with the maximum being reached in the case of large round formations of clogged debris.

**Figure 13:**
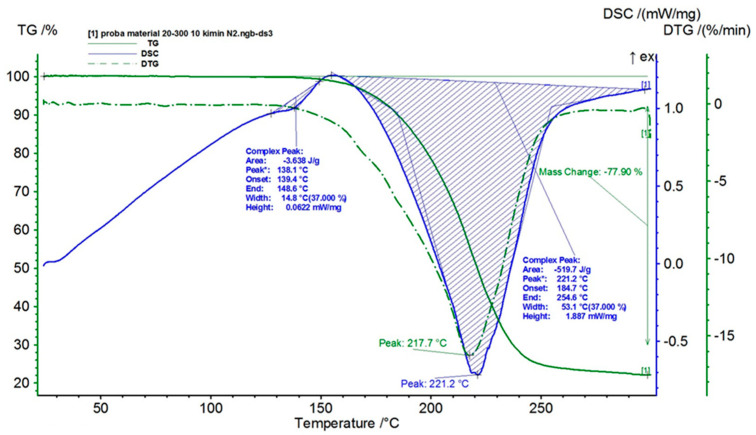
Simultaneous thermal analysis (TG/DSC) of the nanoporous ceramic filter after the 4-week treatment trial-a general overview of the five samples.

The second part of the post-treatment ceramic filter analysis consists of the simultaneous thermal analysis (TG/DSC). These measurements were performed on solid samples of composite polymer materials (5.8 mg), concerning the following parameters: temperature ranging between 20 and 300 °C, heating speed at 10 K/min, working atmosphere of nitrogen, and alumina crucible as the reference substance. Figure 13 shows the variation curves of TG and DSC and of the TG derivative (DTG), depending on temperature (20–300 °C), for the analyzed materials taken from the five samples.

The results obtained from the analysis within the temperature range of 20–300 °C suggest the existence of two endothermic processes: a melting process and a decomposition process. The first process is a low-intensity melting process, which takes place at a maximum temperature of 138.1 °C and presents an enthalpy value of 3.638 J/g. It is known that, depending on the type of protein, the melting point can vary from 85 to 145 °C.

The second process is typical for the decomposition of an organic substance; it has a much higher intensity than the first one, takes place at a maximum temperature of 221.2 °C, and presents an enthalpy value of 519.7 J/g. The total mass loss due to the decomposition process of the analyzed material was 77.90% over the analyzed temperature range. Water-soluble proteins can be included among the organic substances that decompose at this temperature (221 °C).

As a result, both deposition and partial clogging of the ceramic filter with proteins are found, which decompose at temperatures below 300 °C. The ceramic filter’s loading degree is relatively high, almost four times in proportion to the mass. Still, even in these conditions, it was observed via the SEM and EDS analysis that there is not a complete clogging of the pores, but a significant reduction in their global permeability.

## 4. Discussion

In this paper, the authors have presented a laborious way of producing and testing a nanoporous ceramic filter specifically designed for the filtration of a small peptide, Aβ. The nanoceramic filter was the central piece of an experimental CSF filtration device that was implanted in a common AD mouse model as a potential treatment modality. The main aims of the paper were met: the development of a biocompatible Aβ-selective nanoporous ceramic filter, the inclusion of this nanoporous ceramic filter within a prototype used in CSF filtration in an APP/PS1 mouse, and the feasibility of a 4-week trial period of filtration based on indirect evidence from the post-treatment analysis of the ceramic filter.

Based on our previous results [17] and the experience of other research groups [29,30], the optimal procedure for obtaining a high-purity, highly selective nanoporous ceramic filter includes starting with alumina samples processed via a two-step anodizing method in oxalic acid-based electrolytes. After this synthesis, functionalization of the nanoporous ceramic filter is the crucial step in order to obtain the desired porous ceramic filter with the exact nano-characteristics (pore size and shape, and interpore distance). Again, based on our expertise and the most recent finding from the literature [31], we opted for using the ALD technique.

Subsequently, quality control of the synthesis and functionalization occurred. We chose to conduct a thorough check, using many measurement techniques: X-ray diffraction, Raman spectroscopy, infrared spectroscopy, scanning electron microscopy (SEM) with energy dispersive X-ray spectroscopy (EDS), and a simultaneous thermal analysis, as the equipment we have in the laboratory can simultaneously perform thermogravimetry analysis and differential scanning calorimetry. Regarding the spectroscopy measurements, our results align with similar studies conducted on functionalized alumina nanoporous ceramic filters [26,27].

A pleasant surprise was observed after the SEM and EDS testing, as the final parameters of our samples surpassed the preliminary expectations that resulted from the theoretical design. We obtained a nanoceramic filter characterized by a 17 nm pore size, with the pores much more uniform and spatially closer than expected, and with an interpore space of less than 50nm. The simultaneous thermal analysis was also within the expected/desired parameters, confirming the purity of the sample and its lack of organic substance contamination.

Lastly, biocompatibility tests allowed the use of the developed nanoporous ceramic filter for in vivo studies. However, one observation should be made: the increased viability of the two tested cell lines should be interpreted with caution, as the MTT assay, despite being frequently used and most of the time reliable, has its inherent pitfalls and limitations [32].

Both the design and development of the CSF filtration device were inspired by the already in-use CSF shunts successfully employed for hydrocephalus [33]. We modified the standard design to be more filtration-oriented. Thus, our device has three main parts: the catheter, which ensures the connection to the lateral ventricle; the central filtration module, containing the nanoceramic filter; and the reservoir, where Aβ-rich CSF is collected and sequestered based on the reaction between Aβ and anti-Aβ antibodies or Neprilysin–Albumin. The innovations of our proposed model permit a reasonable CSF filtration rate despite the reduced size of the prototype.

Subsequently, the experimental device was implanted in a small group of five APP/PS1 mice, one of the most frequently used AD animal models in fundamental research [34]. Our main objective was to assess the safety of the surgical procedure and post-interventional survival during a 4-week trial period. With a 100% procedural survival rate and no significant adverse effects (i.e., severe infections), our technique seems to minimize the potential associated risks stated in the existing literature [35]. Another considerable goal was the post-treatment assessment of the nanoceramic filter in terms of clogging and possible structural damage. In this sense, the prototypes were explanted from the animals, and the nanoporous ceramic filters were taken out and retested using SEM–EDS and a simultaneous thermal analysis.

The final results indirectly demonstrated the effectiveness of the experimental nanoporous ceramic filter in the filtration of Aβ, as we observed a high degree of protein accumulation, which led to a reduction in pore size (incomplete clogging), thus diminishing the efficacy of the nanoceramic filter. While the accumulated organic residues might also include other proteins besides Aβ, combining size-based filtration with immuno-separation ensures a high degree of Aβ-soluble monomers and oligomers filtration. Considering these findings, the functional lifespan of our designed nanoceramic filter remains around 4 weeks. Theoretically, an extension of the device lifespan could be possible with repeated changes of the nanoporous filter. However, as therapies for NDDs should be long-term, longer-lasting nanoporous ceramic filters with reduced biofouling properties are needed to avoid replacing the filtration devices periodically.

Finally, the limitations of this study should be acknowledged. As explained above, the short functional lifespan of the nanoceramic filter, together with the small number of devices implanted in the AD animal model, remain the main limitations of our experimental model. Other factors that need to be considered are the multitude of steps necessary to obtain and validate the nanoporous filter, each being a potential source of errors. In this regard, future research should try to overcome these highlighted impediments by designing studies of a longer duration, with an increased number of subjects.

## 5. Conclusions

Despite the intensive research conducted in this area, AD, along with the other pathologies forming the group of NDDs, remains a challenge for clinicians. With no current curative drug treatment, minimally invasive methods should be considered. According to current knowledge, Aβ has a critical role in the pathogenesis of AD, with Aβ pathological accumulation in the CNS as the main hallmark of the disease. Moreover, according to several hypotheses, reducing the Aβ cerebral load could be a potentially effective therapeutic approach. Approaching the CSF sink seems more feasible, considering the limitations and drawbacks of surgical procedures at the CNS level.

This study details the most relevant phases of developing and testing a new CSF filtration device, which could be an alternative approach to AD treatment. Developing a device oriented to the selective filtration of Aβ from the CSF could be an essential step toward finding an efficient anti-dementia treatment.

We proposed, designed, and tested a miniaturized prototype to assess this method’s feasibility for an AD model. The critical component of our device is the alumina nanoporous ceramic filter, which was obtained through a laborious, multi-step process. Subsequently, we implanted the device and tested it in vivo over a 4-week trial period. Finally, after the explantation of the device, the nanoporous ceramic filters were assessed. We found a partial reduction in their global permeability, but their basic functionality was still maintained, even though it was reduced by about 75%. Therefore, such advanced nanoporous ceramic filters, with anti-biofouling properties, are feasible candidates to ensure the long-term action of this therapy.

## Figures and Tables

**Table 1:** Most relevant parameters of the functionalized nanoporous ceramic filter.

Parameter	Units	Average Values
Ceramic filter thickness	µm	48
Pore diameter	nm	17 (±1.7)
Pore period	nm	42 (±4.2)
Pore density	1/cm^2^	3.2 × 10^10^ (±20%)
Porosity	%	12
Molecular Weight Cut-Off (MWCO)	kDa	140
(Aβ) Diffusivity	%	100

**Table 2:** Results of the EDS analysis concerning each element and their corresponding composition.

Element	Figure 12a	Figure 12b	Figure 12c	Figure 12d
C	6.62	41.25	70.9	77.68
O	36.82	23.13	9.25	7.23
Na	0	0.04	0.42	0.46
Al	51.43	31.89	14.49	10.22
Si	0.17	0.08	0.46	0.42
P	0.08	0.03	0.61	0.67
S	3.7	2.61	1.85	1.28
Cl	0	0.06	0.72	0.81
Ca	0.04	0.02	0.55	0.36
Au	0.96	0.88	0.77	0.79

## Data Availability

The data that support the findings of this study are available upon request from the corresponding author.
